# Supplementary material for: A computationally frugal, open-source chest CT foundation model for thoracic disease detection in lung cancer screening programmes
Source: Commun Med (Lond). 2026 Feb 4;6:83. doi: 10.1038/s43856-025-01328-1 (PMC12876872; doi:10.1038/s43856-025-01328-1)
Supplement: Supplementary file 1 — Supplemental Material [file 43856_2025_1328_MOESM1_ESM.pdf]

Supplementary Information

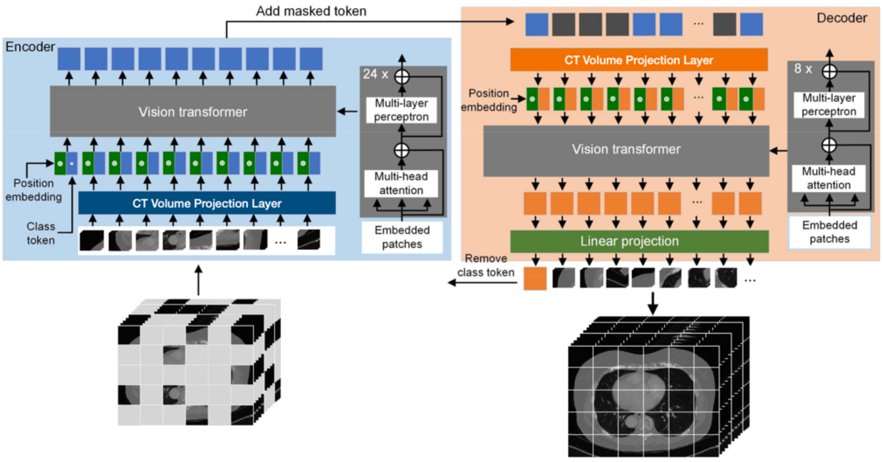

Supplementary Fig. 1 | Overview of the masked autoencoder (MAE) framework used for pretraining TANGERINE. Input CT volumes are divided into 3D patches, with a random subset masked prior to encoding. The encoder processes only the visible (unmasked) patches, reducing memory and compute requirements. Masked tokens are reintroduced before the decoder, which reconstructs the original volume from both masked and unmasked representations. Positional embeddings and CT-specific projection layers are used to preserve spatial context throughout the architecture.

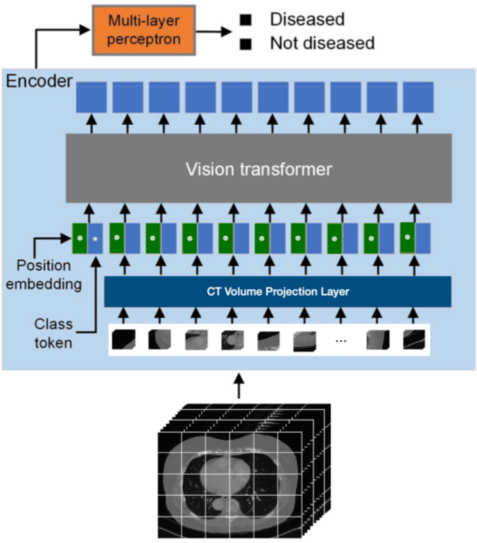

Supplementary Fig. 2 | Downstream fine-tuning architecture for disease classification.
During fine-tuning, only the pretrained encoder is retained from the MAE framework. A multi-layer perceptron (MLP) is added on top of the encoder's output to perform binary classification (e.g., diseased vs. not diseased). Input CT volumes are split into 3D patches, embedded via a CT-specific projection layer, and passed through a vision transformer with positional embeddings and a class token. The model is then fine-tuned end-to-end using labelled data for disease-specific tasks.

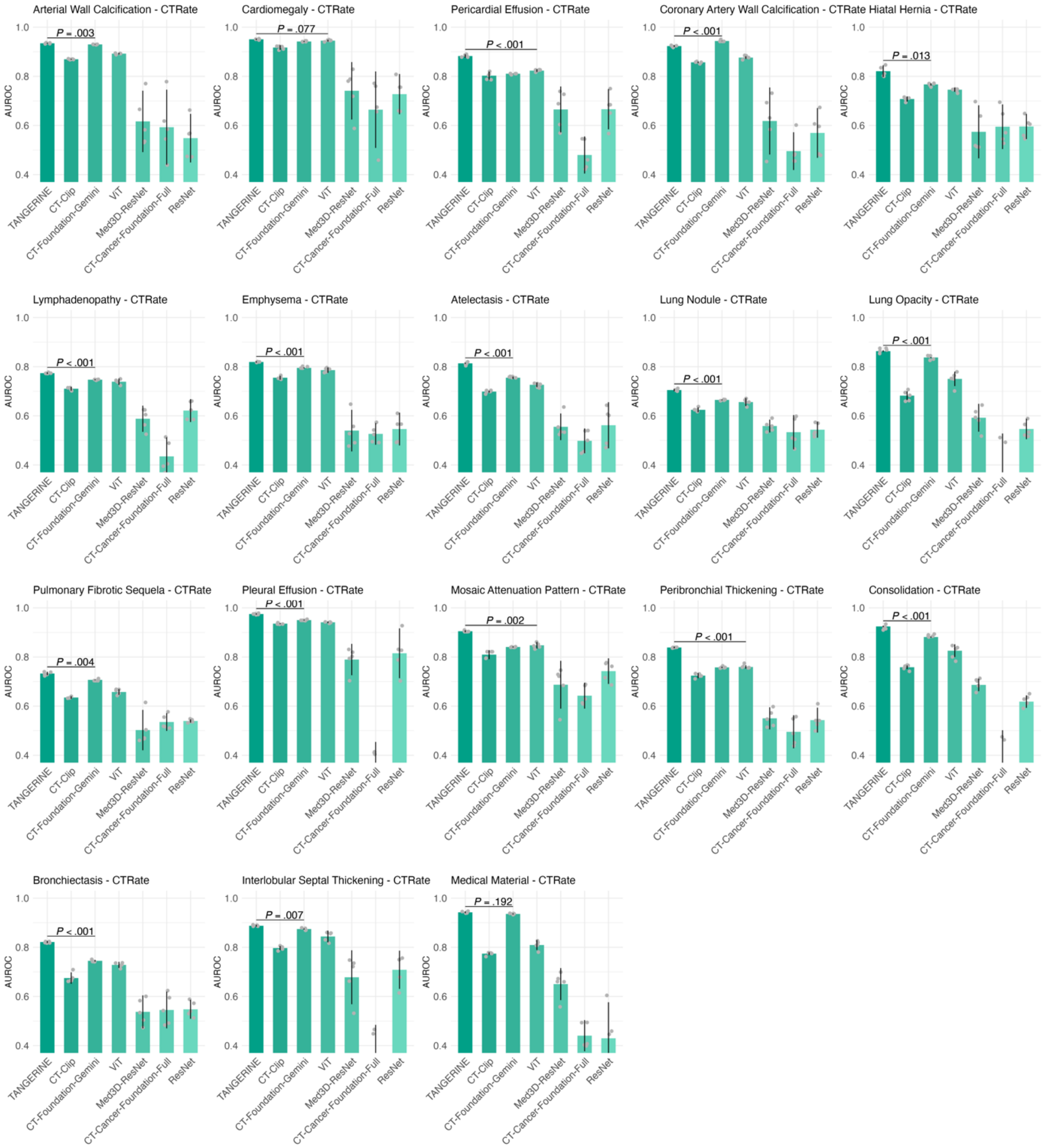

Supplementary Fig. 3 | Performance on CTRate classes in the pretrain-seen-distribution setting. This graph shows the performance of TANGERINE and comparison models on CTRate classes in the pretrain-seen-distribution setting. Models were fine-tuned and evaluated on CTRate, a dataset with distributions exposed to TANGERINE during pretraining. Full dataset sizes, including training, validation, and testing splits are detailed in Supplementary Table 2. Each model was trained with five random seeds; error bars show 95% confidence intervals, and bar centres indicate mean AUROC. Pairwise *P*-values were computed using two-tailed t-tests with Bonferroni correction. The adjusted *P*-values are listed in the figure for the most competitive models (other *P*-values are provided in Supplementary Data 5). TANGERINE demonstrates high performance across CTRate classes in the pretrain-seen-distribution setting.

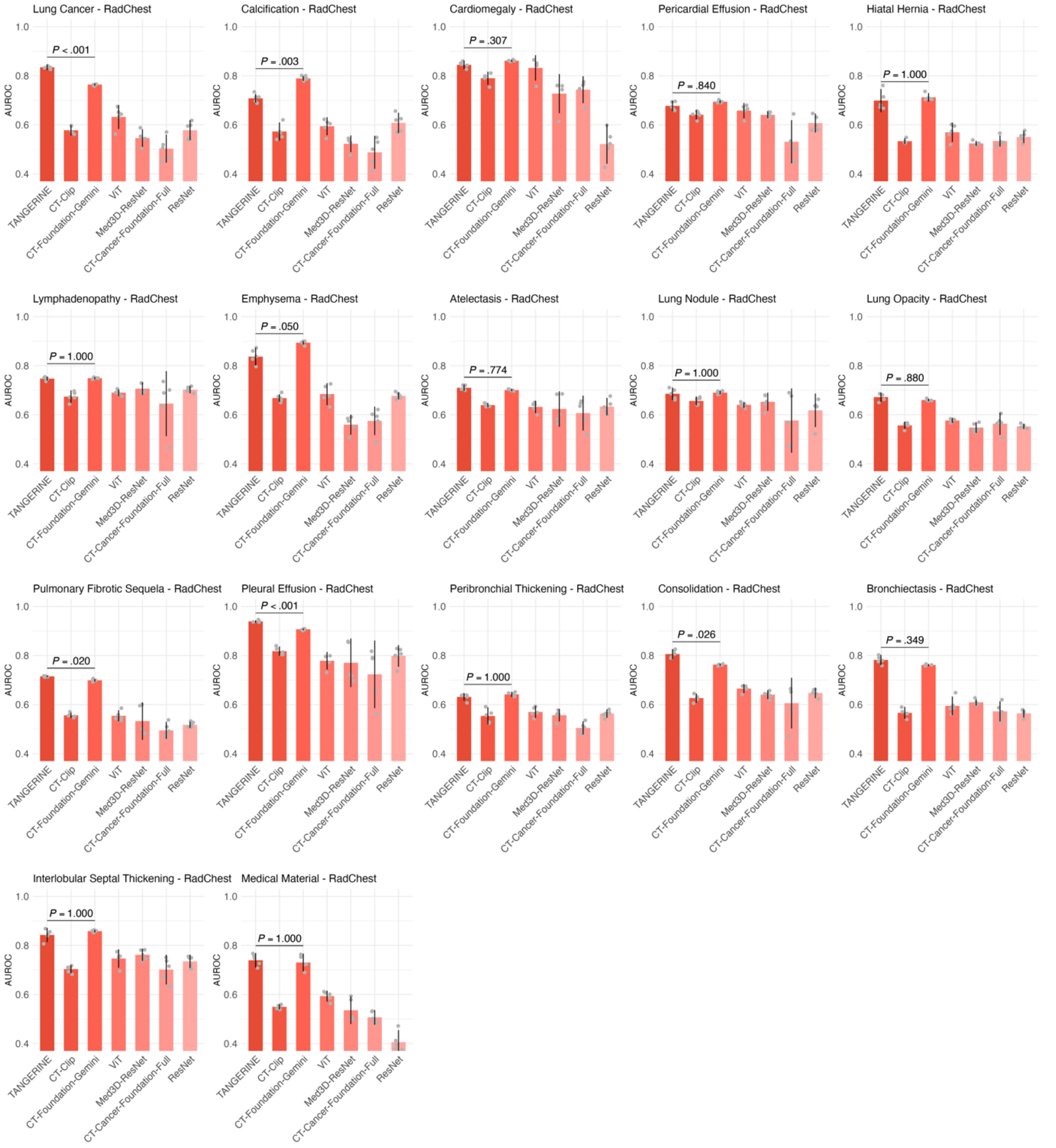

Supplementary Fig. 4 | Performance on RadChest classes in the pretrain-unseen-distribution setting. This graph shows the performance of TANGERINE and comparison models on Radchest classes in the pretrain-unseen-distribution setting. Models were fine-tuned and evaluated on RadChest, a dataset not seen during TANGERINE pretraining. Full dataset sizes, including training, validation, and testing splits are detailed in Supplementary Table 2. Each model was trained with five random seeds; error bars show 95% confidence intervals, and bar centres indicate mean AUROC. Pairwise *P*-values were computed using two-tailed t-tests with Bonferroni correction. The adjusted *P*-values are listed in the figure for the most competitive models (other *P*-values are provided in Supplementary Data 5). TANGERINE demonstrates high performance across RadChest classes in the pretrain-unseen-distribution setting.

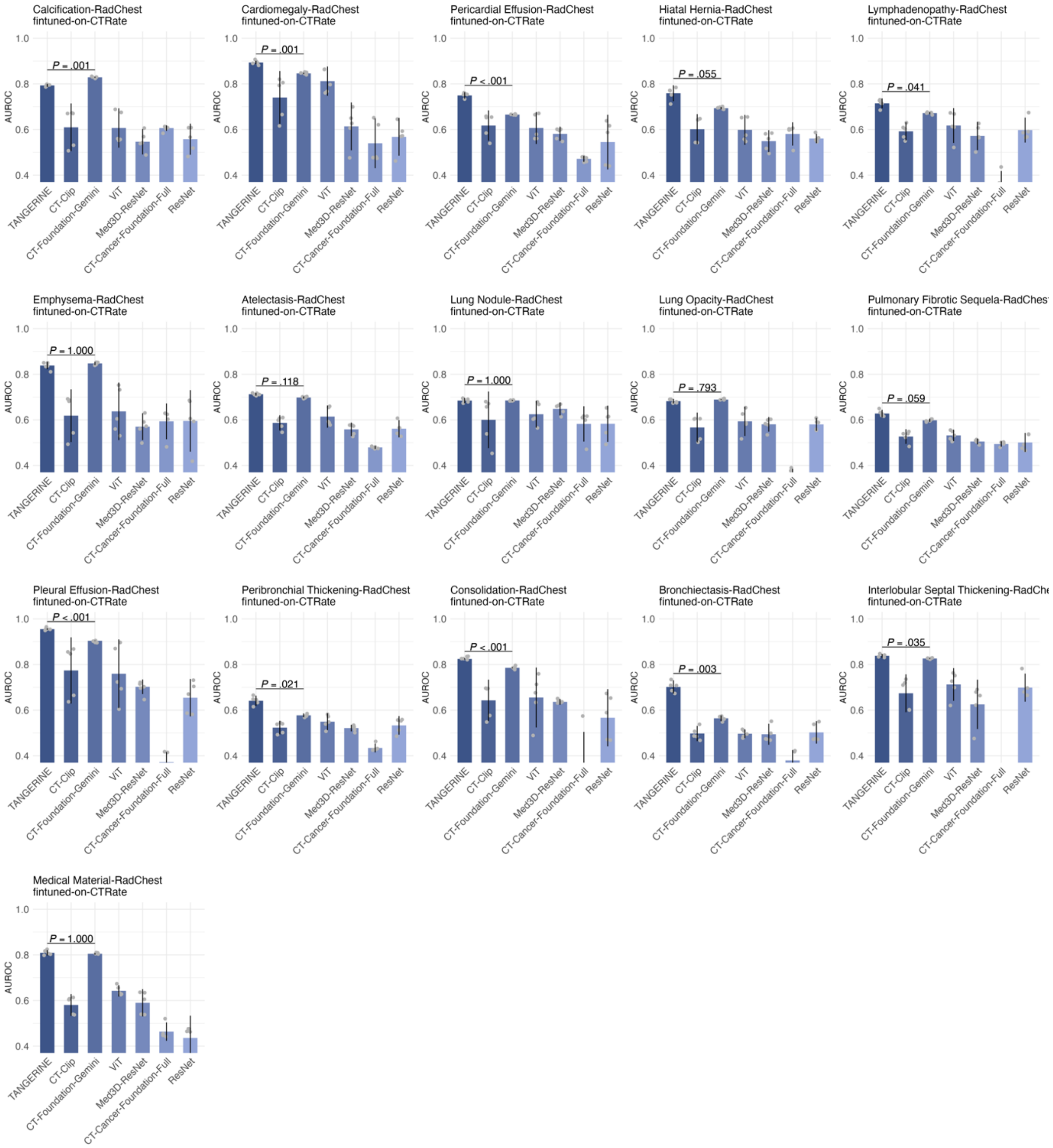

Supplementary Fig. 5 | Performance for domain generalisation on RadChest classes fine-tuned on CTRate. This graph shows the performance of TANGERINE and comparison models on RadChest classes corresponding to class types shared between the RadChest and CTRate datasets. Models were fine-tuned on CTRate and evaluated on RadChest to assess their ability to generalise across domains. Full dataset sizes, including training, validation, and testing splits are detailed in Supplementary Table 2. The error bars represent 95% confidence intervals (CI), and the bar centres represent the mean AUROC. *P*-values were calculated for pairwise comparisons between TANGERINE and all other models using a two-tailed t-test, with Bonferroni correction applied to adjust for multiple comparisons. The adjusted *P*-values are listed in the figure for the most competitive models (other *P*-values are provided in Supplementary Data 8). TANGERINE demonstrates superior or comparable generalisation performance, with robust performance across the shared RadChest classes when fine-tuned on CTRate.

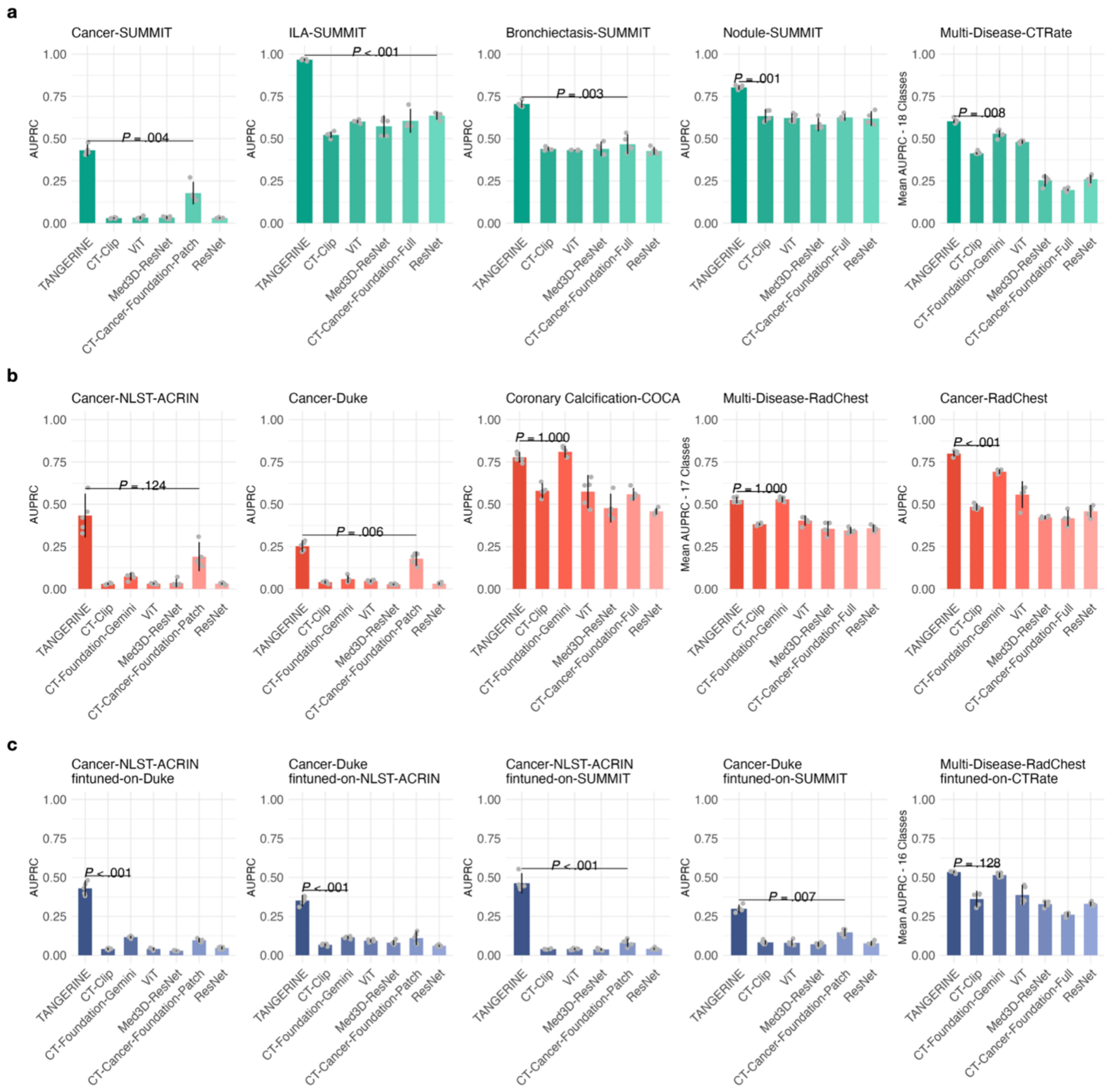

Supplementary Fig. 6 | Performance on lung disease classification – AUPRC. **(a) Pretrain-seen-distribution**: Models fine-tuned and tested on datasets seen during pretraining TANGERINE. **(b) Pretrain-unseen-distribution**: Models fine-tuned and tested on datasets not seen during pretraining. **(c) Domain generalisation**: Models fine-tuned on one dataset and evaluated on a distinct target dataset unseen during pretraining or finetuning. Full dataset sizes, including training, validation, and testing splits are detailed in Supplementary Table 2. Each model was trained with five random seeds; error bars show 95% confidence intervals, and bar centres indicate mean AUPRC. Pairwise *P*-values were computed using two-tailed t-tests with Bonferroni correction (most competitive *P*-values shown, full values in Supplementary Data 7). TANGERINE consistently outperforms or matches comparison models.

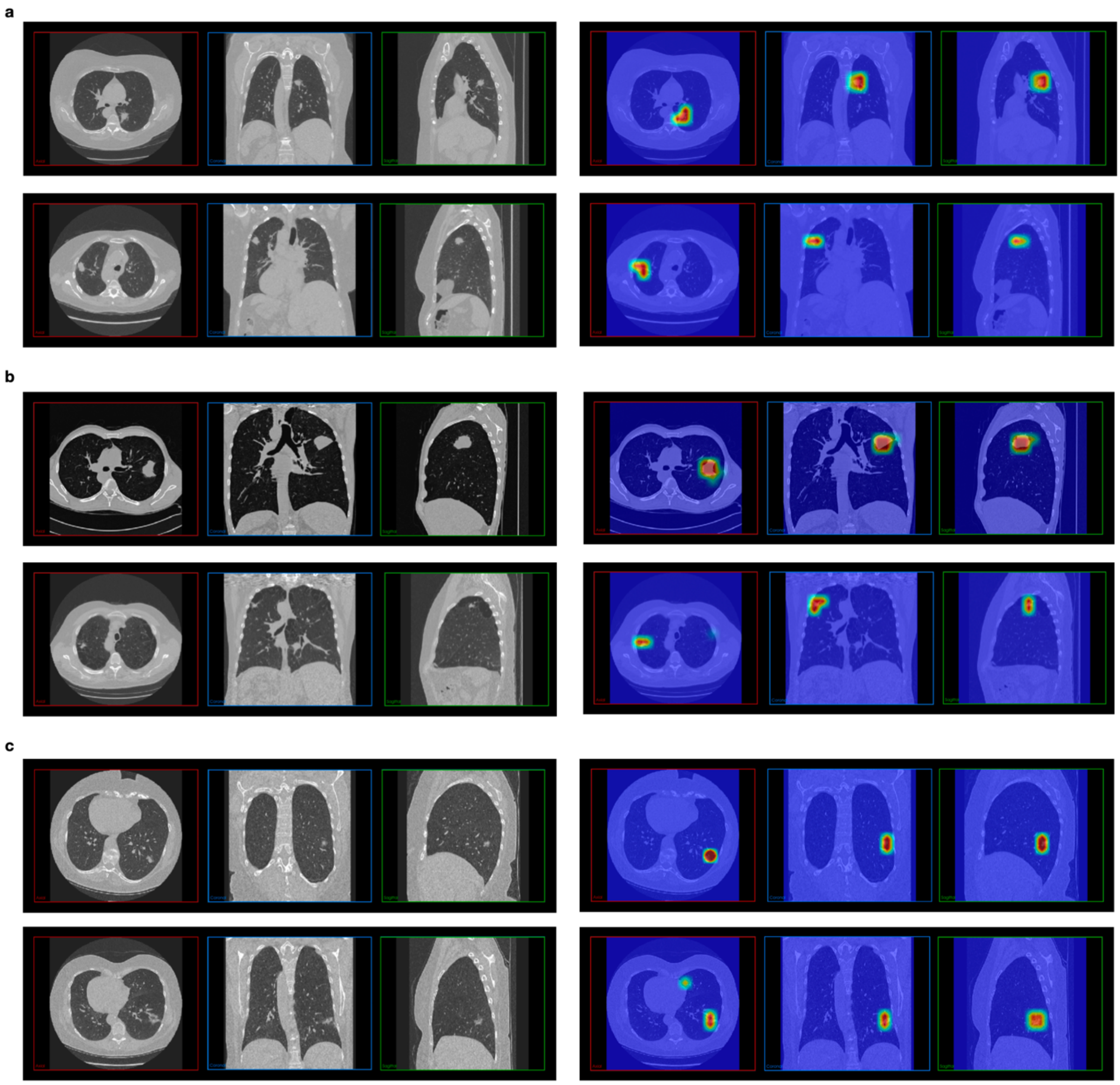

Supplementary Fig. 7 | Cancer classification heatmaps. Example heatmaps illustrating areas contributing to model classifications for various cancer tasks. Red indicates high contribution. On the left are the original scans in axial, coronal and sagittal order, while on the right are the overlaid Grad-CAM heatmaps for: **(a)** Duke, **(b)** NLST-ACRIN, and **(c)** SUMMIT. The highlighted cancerous tumour locations demonstrate that the model predictions are anatomically consistent.

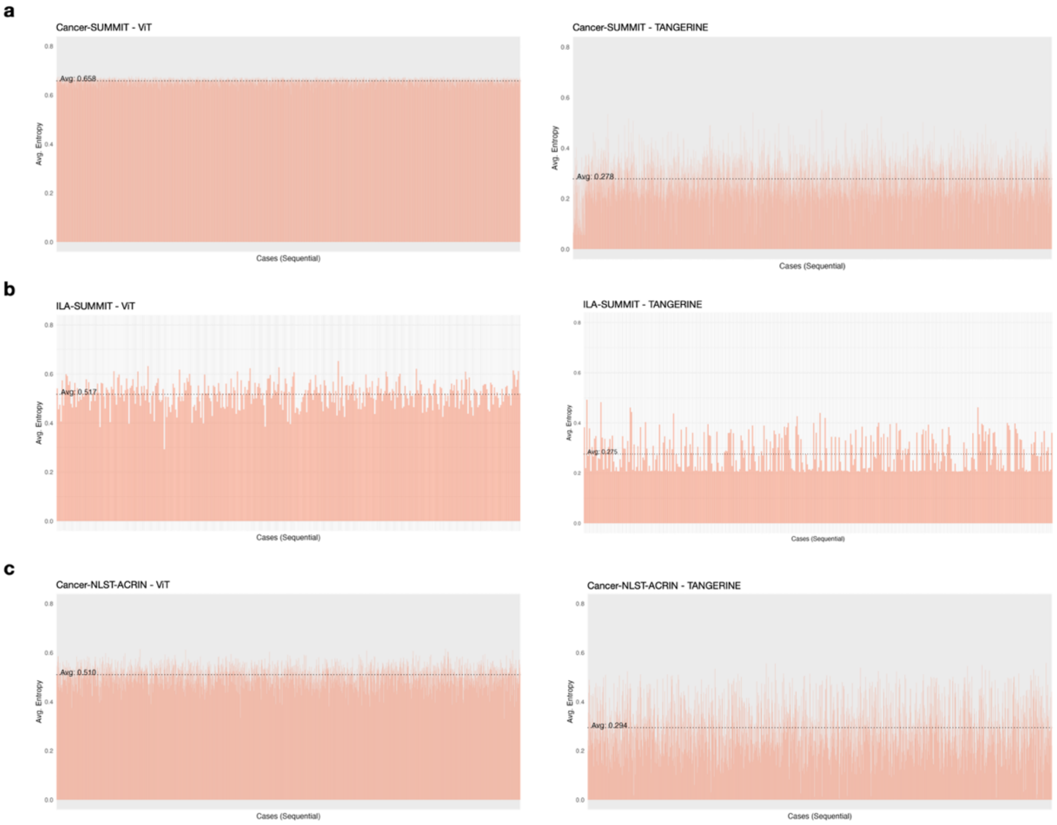

Supplementary Fig. 8 | Uncertainty quantification across datasets and models using entropy. For each dataset **(a) Cancer-Summit**, **(b) ILA-Summit**, and **(c) NLST-ACRIN** the bar plots show the average entropy per case after applying aggressive test-time augmentation (n=50 predictions per case). Results are presented for two models: ViT (left) and TANGERINE (right). The dotted line indicates the average entropy across all cases for each model and dataset. These visualisations highlight the distribution of uncertainty in model predictions under augmented conditions, with higher entropy reflecting greater uncertainty. Full dataset sizes, including training, validation, and testing splits are detailed in Supplementary Table 2.

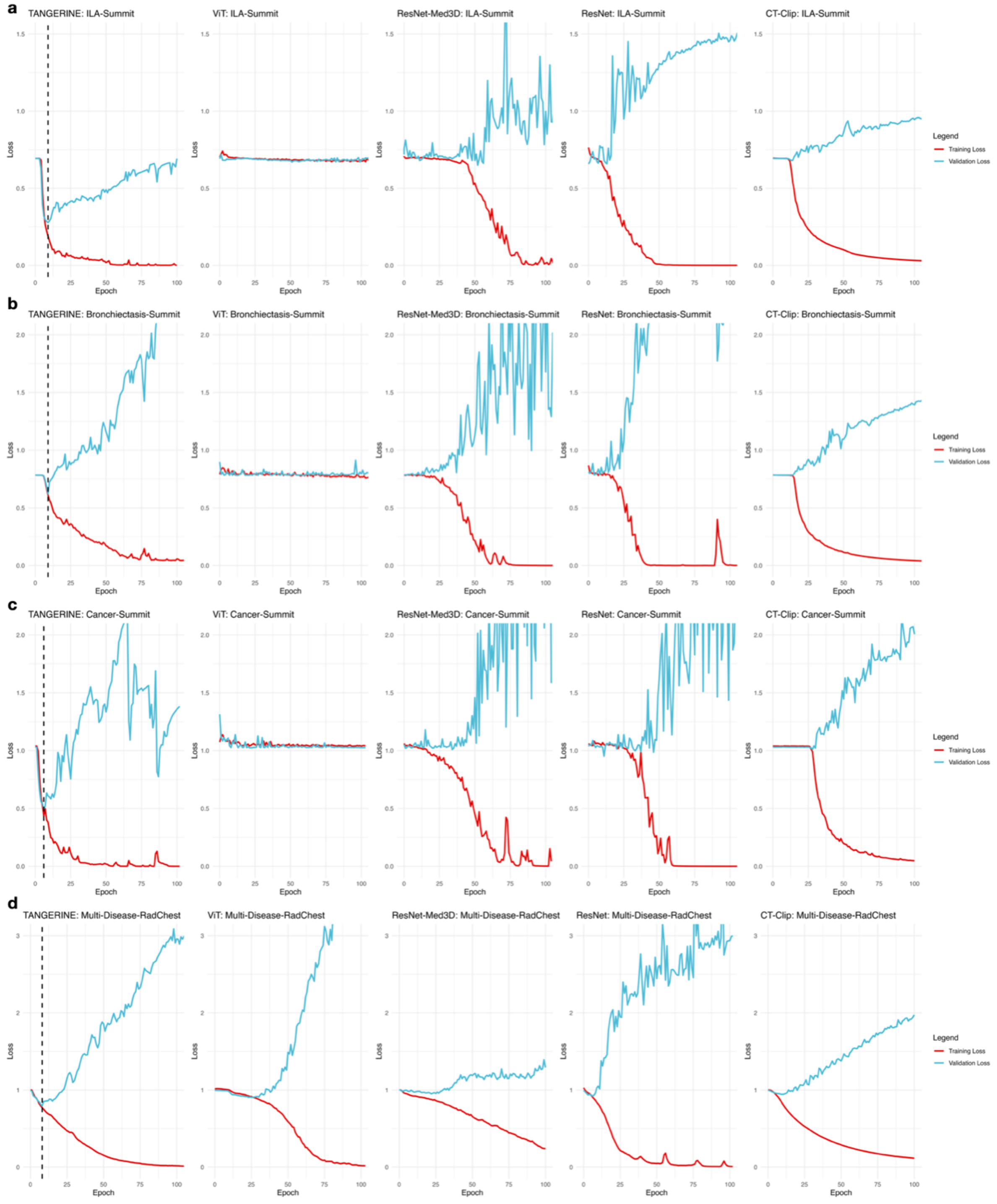

Supplementary Fig. 9 | Training and validation loss curves for example fine-tuning tasks. (a) ILA-SUMMIT. (b) Bronchiectasis-SUMMIT. (c) Cancer-SUMMIT. (d) Multi-Disease-RadChest. First 100 epochs of training and validation loss curves are shown for TANGERINE and comparison models across example fine-tuning tasks. TANGERINE consistently attains a lower validation loss, indicating increased robustness and generalisation capability. While comparison models converge in terms of training loss, their validation loss fails to decrease suggesting the models fail to learn pattens which are generalisable beyond the training set. This pattern highlights TANGERINE's ability to achieve more stable performance beyond the training set, maintaining better generalisation compared to comparison models. The dotted line indicates TANGERINE optimal epoch for early stopping based on lowest attained validation loss. Full dataset sizes, including training, validation, and testing splits are detailed in Supplementary Table 2.

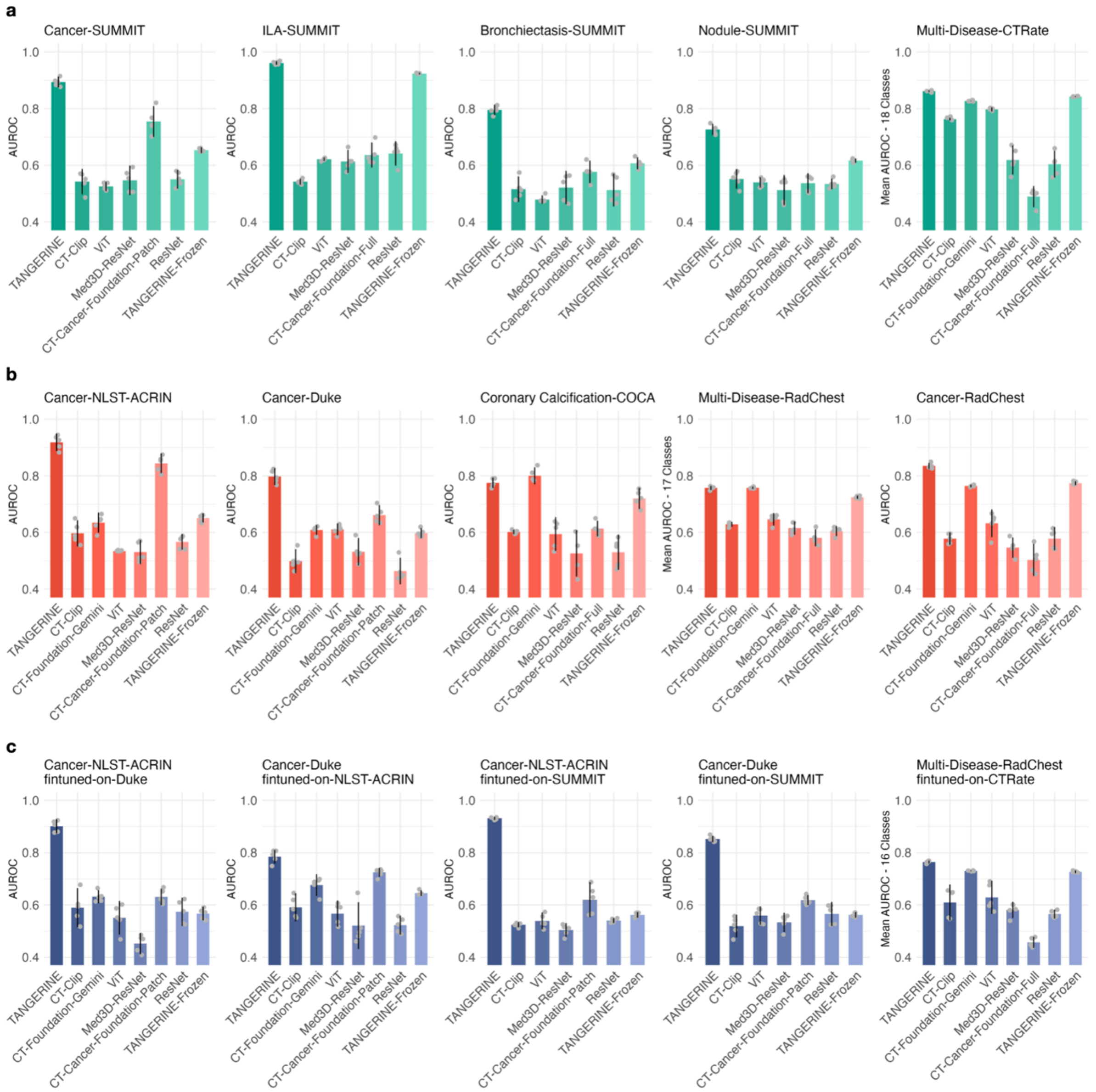

Supplementary Fig. 10 | Performance on lung disease classification with TANGERINE embeddings. (a) Pretrain-seen-distribution: Models fine-tuned and tested on datasets seen during pretraining TANGERINE. **(b) Pretrain-unseen-distribution**: Models fine-tuned and tested on datasets not seen during pretraining. **(c) Domain generalisation**: Models fine-tuned on one dataset and evaluated on a distinct target dataset unseen during pretraining or finetuning. Full dataset sizes, including training, validation, and testing splits are detailed in Supplementary Table 2. Each model was trained with five random seeds; error bars show 95% confidence intervals, and bar centres indicate mean AUROC. Use of frozen TANGERINE embeddings (TANGERINE-Frozen) yields strong performance relative to comparison models, with further improvements achieved through end-to-end fine-tuning (TANGERINE).

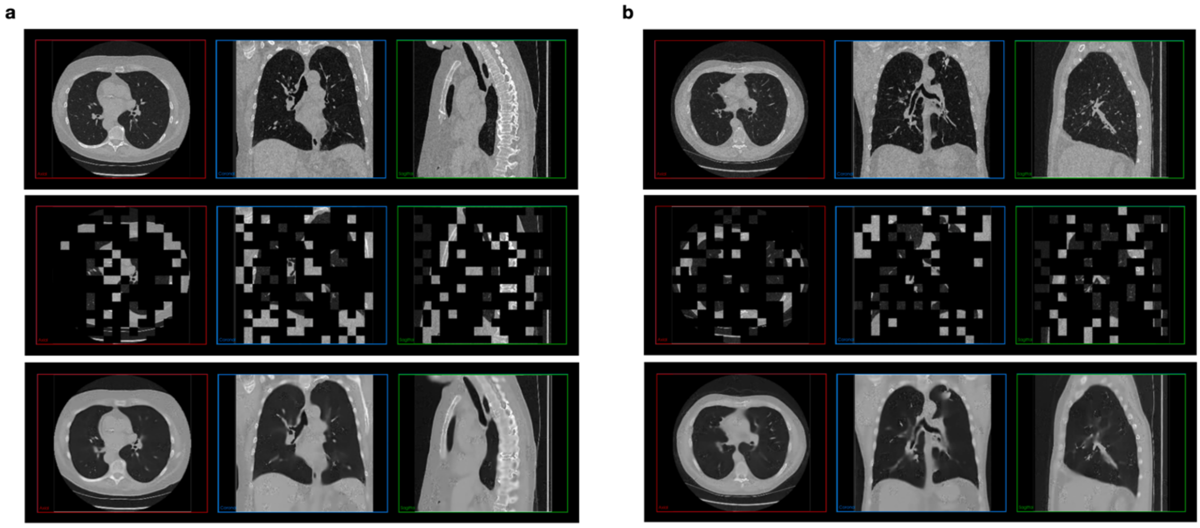

Supplementary Fig. 11 | Example reconstructions from the masked autoencoding pretraining task. Representative axial, coronal, and sagittal CT slices before masking (top row), after random masking (middle row), and after reconstruction by the TANGERINE encoder-decoder model (bottom row). Despite heavy masking across multiple anatomical planes, the model accurately reconstructs key lung structures, including bronchi, blood vessels, and pleural boundaries, highlighting the model's capacity to learn spatially coherent representations critical for downstream thoracic disease tasks.
