# Supplementary material for: A computationally frugal, open-source chest CT foundation model for thoracic disease detection in lung cancer screening programmes
Source: Commun Med (Lond). 2026 Feb 4;6:83. doi: 10.1038/s43856-025-01328-1 (PMC12876872; doi:10.1038/s43856-025-01328-1)
Supplement: Supplementary file 2 — Description of Additional Supplementary Files [file 43856_2025_1328_MOESM2_ESM.pdf]

Description of Additional Supplementary Files

Supplementary Data 1: Pretraining Datasets

Supplementary Data 2: Downstream Tasks Datasets

Supplementary Data 3: Results - Binary Downstream Tasks

Supplementary Data 4: Results - Multi-Label RadChest

Supplementary Data 5: ResultsMulti-Label CTRate

Supplementary Data 6: MultiLabel-RadChest fine-tuned on CTRate

Supplementary Data 7: AUPRC

Supplementary Data 8: Label Efficiency Results-Binary Downstream Tasks using 40% fine-tuning data

Supplementary Data 9: Label Efficiency Results-Multi-Label RadChest using 40% fine-tuning data

Supplementary Data 10: Label Efficiency Results: Multi-Label CTRate using 40% fine-tuning data

Supplementary Data 11: Label Efficiency Results - Binary Downstream Tasks using 10% fine-tuning data

Supplementary Data 12: Label Efficiency Results-Multi-Label RadChest using 10% fine-tuning data

Supplementary Data 13: Label Efficiency Results-Multi-Label CTRate using 10% fine-tuning data

Supplementary Data 14: Comparing label-efficiency - Binary Classification - TANGERINE (40% fine-tune data) vs comparison models (100% fine-tune data)

Supplementary Data 15: Comparing label-efficiency TANGERINE (10% fine-tune data) vs comparison models (100% fine-tune data)

Supplementary Data 16: Comparing label-efficiency RadChest TANGERINE (40% finetune data) vs comparison models (100% fine-tune data)

Supplementary Data 17: Comparing label-efficiency - RadChest: TANGERINE (10% fine-tune data) vs comparison models (100% fine-tune data)

Supplementary Data 18: Comparing label-efficiency - CTRate: TANGERINE (40% finetune data) vs comparison models (100% fine-tune data)

Supplementary Data 19: Comparing label-efficiency - CTRate: TANGERINE (10% finetune data) vs comparison models (100% fine-tune data)

Supplementary Data 20: Binary Downstream Tasks - Different Pretrain Dataset Sizes

Supplementary Data 21: RadChest - Different Pretrain Dataset Sizes

Supplementary Data 22: CT-Rate - Different Pretrain Dataset Sizes

Supplementary Data 23: MultiLabel-RadChest finetuned on CTRate - Different Pretrain Dataset Sizes

Supplementary Data 24: Pretrained Model Weights
